# Establishing a minimum PMI for bone sun bleaching in a UK environment with a controlled desert-simulated comparison

**DOI:** 10.1007/s00414-020-02385-y

**Published:** 2020-08-15

**Authors:** Sarah Stokes, Nicholas Márquez-Grant, Charlene Greenwood

**Affiliations:** Cranfield Forensic Institute, Cranfield University, Defence Academy of the United Kingdom, Shrivenham, SN6 8LA UK

**Keywords:** Forensic taphonomy, Forensic anthropology, Bone bleaching, FTIR, Bone weathering

## Abstract

Microenvironments play a significant part in understanding the post-mortem interval in forensic taphonomy. Recently, the value of weathering factors in relation to obtaining a PMI has been investigated further. In this study, observations were made to calculate the length of time it takes for three different bone elements (femur, rib, and scapula) to bleach in a UK summer and winter. This research also investigated whether there were any physicochemical modifications to the bone caused by bleaching. Porcine femora, scapulae, and ribs were placed into open and shaded areas of an outdoor research facility located in Oxfordshire, UK, during summer (July–Sep) and winter months (Dec–Mar). The specimens were monitored at 3-week intervals using photography, and an observational scoring method was developed to quantify the extent of bleaching. As temperatures are typically much lower in the UK compared with warmer climates, a controlled indoor-simulated desert experiment was also undertaken to be used as a control. This allowed sun bleaching and changes to the bone chemistry to be monitored in a controlled, high-UV environment for comparison with the UK outdoor experiments. Fourier transform infrared spectroscopy (FTIR) was employed to analyze physicochemical modifications to both the mineral and organic components of the bone. The FTIR was used to calculate crystallinity index (CI), mineral to organic ratio, and the relative amount of carbonate concentrations. Weather data was collected and a positive correlation was found between both ultraviolet (UV) levels and accumulated degree days (ADD) when compared with observational bleaching scores. Bleaching (whitening) of the bone samples occurred in both seasons but at different rates, with the bleaching process occurring at a slower rate in winter. During summer, the initial bleaching process was evident at 6 weeks, and by 9 weeks, the bones were an off-white colour. During the winter period, whitening of the bone started at 9 weeks; however, only the scapula and rib samples displayed a similar off-white colour. This colouration was observed at 13 weeks rather than at 9 weeks. The desert simulation samples started bleaching in a similar pattern to the outdoor samples after 1 week but the bones did not fully bleach. The bone chemistry, based on physicochemical properties obtained from the FTIR, showed a significant statistical difference between the simulated desert and winter season when compared against a control sample. For the winter samples, the mineral to organic ratio was significantly higher than that in the control, suggesting a reduction in the proportion of organic. For the samples in the simulated desert environment, the crystallinity index was significantly higher than that in the control samples, suggesting an increase in crystallinity. The results of this experiment support the fact that it is possible to achieve bleaching in a UK environment and that the minimal time frame for this to occur differs in seasons.

## Introduction

Taphonomy started as a subdiscipline of palaeontology. Palaeontologists studied the appearance and condition of fossilized skeletal remains, its name originating from the Russian Scientist Yefremov in 1940 [1–4].

In recent years, there has been an increase in research focused on bone weathering to assist with minimum post-mortem interval. Weathering stages for bone have been attributed as being introduced by Behrensmeyer in 1978 whereby changes were noted through years of exposure [5]. Haculak and Rogers [6], Janjua and Rogers [7], Myburgh et al. [8], Ross and Cunningham [9], and Schultz et al. [10] investigated developing PMI models based on bone erosion and colouration in a number of environments. Apart from the reporting on observations made on forensic cases, forensic taphonomy research has also recently been developed further by introducing outdoor research facilities to monitor the effects of specific environments [11].

As establishing a minimum post-mortem interval (PMI) can be extremely challenging, recent studies have been carried out to assist with identifying the variable effects that contribute to the alteration and decomposition of the body after death [1, 5–10]. Andrews and Cook [12] studied the decomposition of a cow in a woodland area in Somerset, England. The bones had not reached a weathering stage beyond stage 0. In 1978, a study performed in Wales had also concluded that no weathering had taken place even after 10–12 years. In warmer climates such as South America, weathering stages are absent as well as bleaching even after many years if the remains are buried [9].

Wang et al. [13] analyzed 112 skull samples (buried and unburied) and quantified changes to the organic component of the bone with increasing PMI using FTIR and suggested that a reliable method could possibly be developed and used in the future. However, this study was aimed to identify chemical changes between rates of decomposition to gather an estimated PMI, as oppose to bleaching of samples.

Bleaching occurs when the ultraviolet rays from the sun breaks down the bonding of the molecules of an object and in a process known as photodegradation; the colour of the object fades [12–14]. Previous research on bone bleaching suggests that sunlight is not the only cause for discolouration as it has also been reported to occur in marine environments. Pokines and Higgs [15] examined twenty-five samples of various human bones from the ocean and examined a variety of taphonomic factors that had taken place, including bleaching. It was found that 88% of the samples showed evidence of bleaching having occurred in the marine environment. This was found to be due to the breakdown of the organic components.

In 2017, Schulz and colleagues developed a study with an aim to progress Behrensmeyer’s weathering stages to evaluate a methodology for bone bleaching and establish a PMI interval. The study was based in Florida where colour changes were observed which confirmed that weathering data can be used to estimate a PMI. Early signs of bleaching were noted to occur much sooner than shaded samples, and these were monitored using an observational scoring system that incorporated the Behrensmeyer’s weathering stages [10].

Colour changes have also been proven to occur when a bone is heated. In 2014, Greenwood studied physicochemical modifications to bone mineral. It was concluded that with a significant increase in temperature, bone colour would turn white due to calcination. This also occurred when the bone is left in high temperatures over a period of hours [14].

There is currently limited scientific information regarding bone bleaching within a UK environment in forensic taphonomy. The main aim of this research was to establish whether a time estimate (minimum PMI) can be calculated for the time it takes for a bone to bleach based on visual and physicochemical properties of the bone. This was achieved by exposing bone to a sun exposed surface, a shaded area and controlled environments during the seasons of summer and winter in the UK. Additionally, a simulated desert experiment was included as a control in the experiment. Artificial UV lighting was used to allow for bleaching comparison as UV levels are found to be much higher in warmer climates. The physiochemical properties of the bone were also investigated using Fourier transform infrared spectroscopy (FTIR) in order to investigate any chemical changes to the bone.

If a length of time can be established for when bleaching occurs, it could assist with providing a minimum PMI in cases where bone has been found, for example, in an open field in a similar environment within the UK.

## Materials and methods

### Materials

Porcine (*Sus scrofa domesticus*) bone was utilized for this experiment. Fresh porcine samples were collected from a local abattoir (Staffordshire Meat Packers, UK). These samples were fresh on the day of collection and immediately taken to the laboratory at Cranfield University, Defence Academy of the United Kingdom, Shrivenham, UK. The pigs were all aged 4 to 6 months old and were bred for produce at the abattoir farm. There were twelve femora, twelve scapulae, and twelve individual ribs that were sampled for both summer and winter seasons. A scalpel was used to deflesh the bone of all soft tissue. Once defleshed, the bones were rinsed with de-ionized water. Five of each bone type were cut into approximately 3 × 0.5 × 0.5-cm fragments to allow for chemical analysis (analytical samples) whilst six of each type were kept complete for observational analysis (observation samples). Control samples (fresh frozen bone) from each bone element were obtained for comparison. These control samples were used for chemical analysis. One of each bone type of both observational and analytical samples was placed under shade. All the samples and fragments were weighed before and after the experiment. At the end of the observational study, the samples were stored in an – 18 °C freezer and thoroughly thawed prior to the FTIR analysis.

### Methods

Long bones (femur) and flat bones (scapular and ribs) were sampled to analyze differences between types of bones if bleaching was to occur.

Weather data was collected daily between the summer months of 21 June 2017 and 22 September 2017; and the winter months between 21 December 2017 and 22 March 2018. UV levels, temperature, precipitation, humidity, and wind velocity were collected at three points during the day (6 am, 12 pm, and 6 pm) for the entire length of both seasons using the online weather database (Accuweather). The weather data that was collected was used to explore a correlation coefficient by measuring the UV and calculating accumulated degree days (ADD) and examining if there is a pattern of correlation against bleaching levels.

The samples were weighed (g), and the cortical thickness of the samples was measured before and after exposure to the outdoor environment to observe mass reduction. Photographs were taken at each interval to document the observational findings and record changes in the positioning, colouring, and weathering of the bones.

Chemical analysis was carried out using a Bruker Alpha Platinum ATR FTIR. A scan resolution of 4 cm^−1^ and 16 scans were employed for data collection, within a range of 2500–400 cm^−1^. Physicochemical modifications to the mineral and organic components of the bone were investigated for the control and experimental samples. Spectragryph 1.2 was used to calculate the area of the *v*_3_ phosphate (1200–900 cm^−1^), amide (1750–1600 cm^−1^), and *v*_3_ carbonate (890–850 cm^−1^) bands. Carbonate to phosphate and amide I to phosphate ratios were calculated to assess changes to the carbonate within the bone mineral and collagen, respectively, and the splitting factor *v*_4_ was calculated to assess changes to the crystallinity index. These physicochemical variables were used to compare control samples against experimental samples at different intervals of sun exposure to confirm whether bone changes had taken place due to bone bleaching.

A CM700d spectrophotometer was used to analyze the colour space values (L*A*B) to determine whether a pattern of colour change can be quantified. These were taken with an aim to obtain a fixed scientific value for the colour changes that were being observed.

To monitor visual colour changes, an observational scoring system was developed. For each section of bone, a score of 1–5 (number value) was given at each 3-week interval, 5 being complete white and 1 being a natural bone colour. A fully bleached bone equated to an ‘off-white to white’ colouration with no areas of brown or yellow staining across the entirety of the bone. This allowed for quantitative measure.

Thus, the bone colour bleaching score was assessed on the following:

1. Natural bone colour—beige with a tint of brown
2. Cream—cream beige in colour with tints of yellowing
3. Off-white—cream with no tints of yellow or brown
4. Brilliant white—bright white
5. Desert white—–bright white with drying/flaking

Sections of bone were observed on their coloured appearance and given an observational score between 1 and 5 as above. The scores were then added to give a total for each bone. The sections of bone that were scored were as follows:

*Femur—*medial and lateral condyle, patellar surface, medial and lateral epicondyle, medial and lateral distal shaft, anterior and posterior distal shaft, medial and lateral proximal shaft, anterior and posterior proximal shaft, anterior and posterior mid-shaft, medial and lateral mid-shaft, and the head and neck

*Scapular—*supraspinatus fossa, infraspinatus fossa, posterior surface area, glenoid cavity, spine, and neck

*Rib—*external shaft, internal shaft, and distal and proximal rib ends

#### Analytical sample preparation

A total of 30 porcine femur samples were randomly selected for chemical analysis. Five bleached samples from the summer, the simulated desert, and from the winter experiment were taken as well as five control samples. The sections were washed with de-ionized water and cut into 1-mm slices using a diamond cutting blade. The slices were then milled into a fine powder using a Retsch mixer mill and sieved using 106-μm mesh sieve. Bone colour was assessed using CIE (International Commission on Illumination) L*A*B colour scale with a CM700d spectrophotometer.

#### Outdoor taphonomy facility

The samples were placed in a supine position at a taphonomy facility at Cranfield University Shrivenham. The facility was approximately 90 m^2^ with a 1.5-m highly meshed fence. For the open sun-exposed area, the area was approximately 6 m^2^. Samples that were placed over the summer period were put out on the first day of summer (21 June 2017) and were collected on the last day of the season (22 September 2017). This method was also employed for the winter samples. A chicken mesh fencing was placed around this area to reduce scavenging. The soil was a light sedimentary sandy type. The soil pH was taken before and after the experiment. A wooden board was placed over an area of grass and covered with soil from the surrounding area which allowed the bones to be at the same level of UV exposure and reduced the green algae staining that is often found on bleached bone in these types of environments [1, 6]. The shaded area was covered with a canopy of approximately 6 m^2^. Additionally, a plastic crate was used to cover the samples to minimize exposure to sunlight, which ensured temperature regulation and ventilation. Photographs and sketches were taken at each collection to assess the level of bleaching.

#### Simulated desert environment

Additionally, a high-UV environment that replicated a desert was created and placed within the laboratory to see if higher UV levels have greater impact on bleaching than the expected levels observed within the UK. An Arcadia 10% UVA UVB reptile light bulb was used for UV radiation, with a UV index of approximately 9–10. For further information on UV indexes, refer to [16]. For observational analysis, seven femora and seven rib samples were utilized for this experiment, with no scapulae. For chemical analysis, 24 femora and 24 rib fragment samples were placed inside the tank. The samples were placed on Prorep Leo Life reptile sand, and a 100-w heat lamp was placed into the tank to replicate the infrared temperatures. Samples were placed inside a reptile tank for a period of 8 weeks, with the fragments examined every week.

Control samples were placed in a supine position into a box, on a layer of sand and placed in a dark cupboard where there was limited access to UV exposure.

### Limitations

The outdoor facility was situated in an open-field area to collect accurate bleaching sun exposure. The limitations of exposing the bones to the elements are that it was not possible to control other weathering factors that could have interrupted the experiment. This became noticeable during winter when storms were present, disrupting the position of the samples. Precipitation would also have affected the positioning of the bones on the platform. Scavenging was another limitation as although the samples had been fenced, some samples were taken during the winter months. This affected the overall reliability of the results and made it difficult to assess the percentage of bleaching that occurred out of all samples.

The limitations for the simulated desert environment were the reliability of the equipment. The UV lighting could have been better equipped. The UV bulb that was used was designed for reptile tanks and so did not reach an expected peak UV reading after the first interval that would be expected in the desert. As the samples were situated in a tank, they did not get the same outdoor exposure to other weathering factors that would occur in the desert, such as colder night temperatures, rain, or sandstorms.

Porcine (*Sus scrofa domesticus*) bone was utilized for this experiment for ethical purposes. A limitation of this is the microstructural bone differences between human and pig [17]. However, porcine has provided a firm foundation for research in taphonomy for many years and a recent comparative study [18] concluded porcine to be an adequate substitute when human bone is difficult to obtain.

## Results

### Observational analysis

**Fig. 1:**
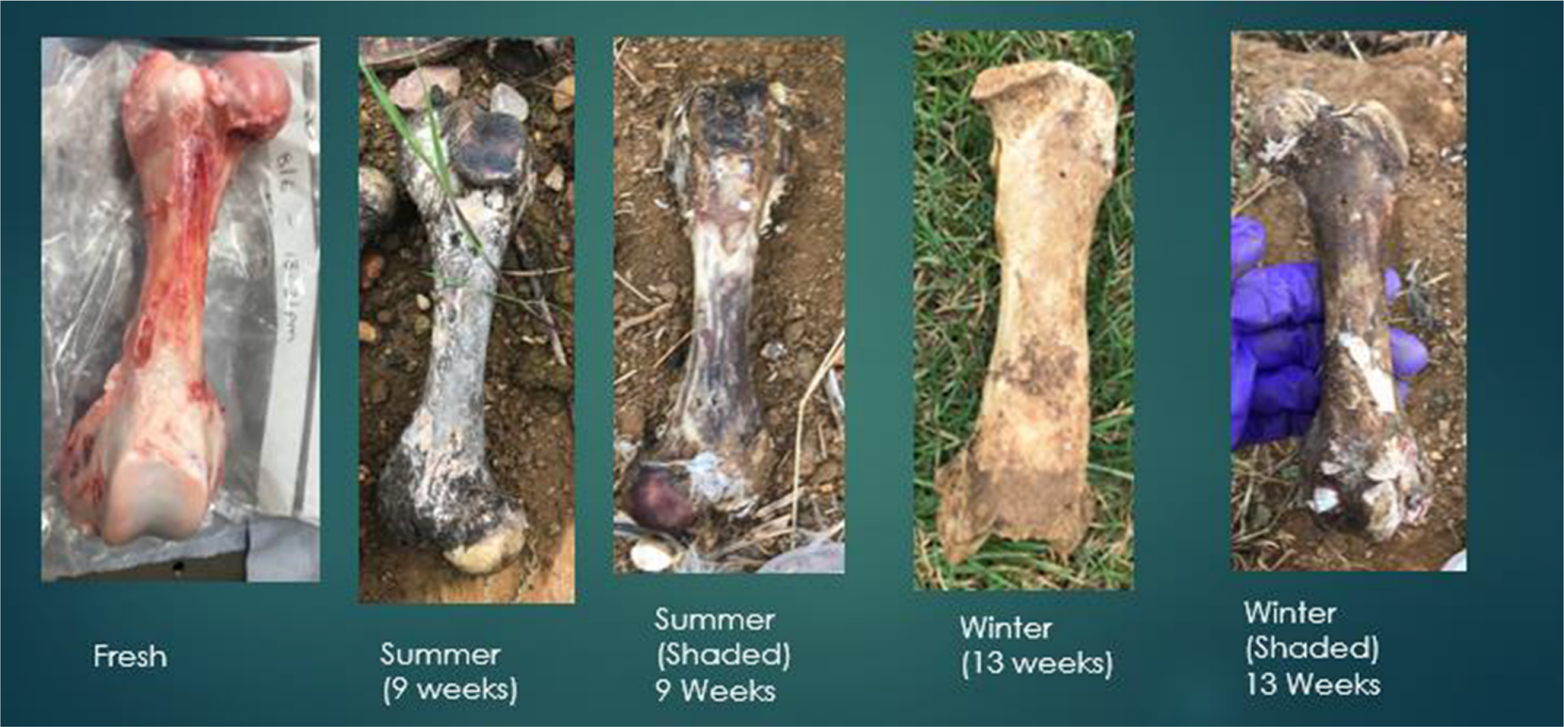
Colour changes to femora at different observations

**Fig. 2:**
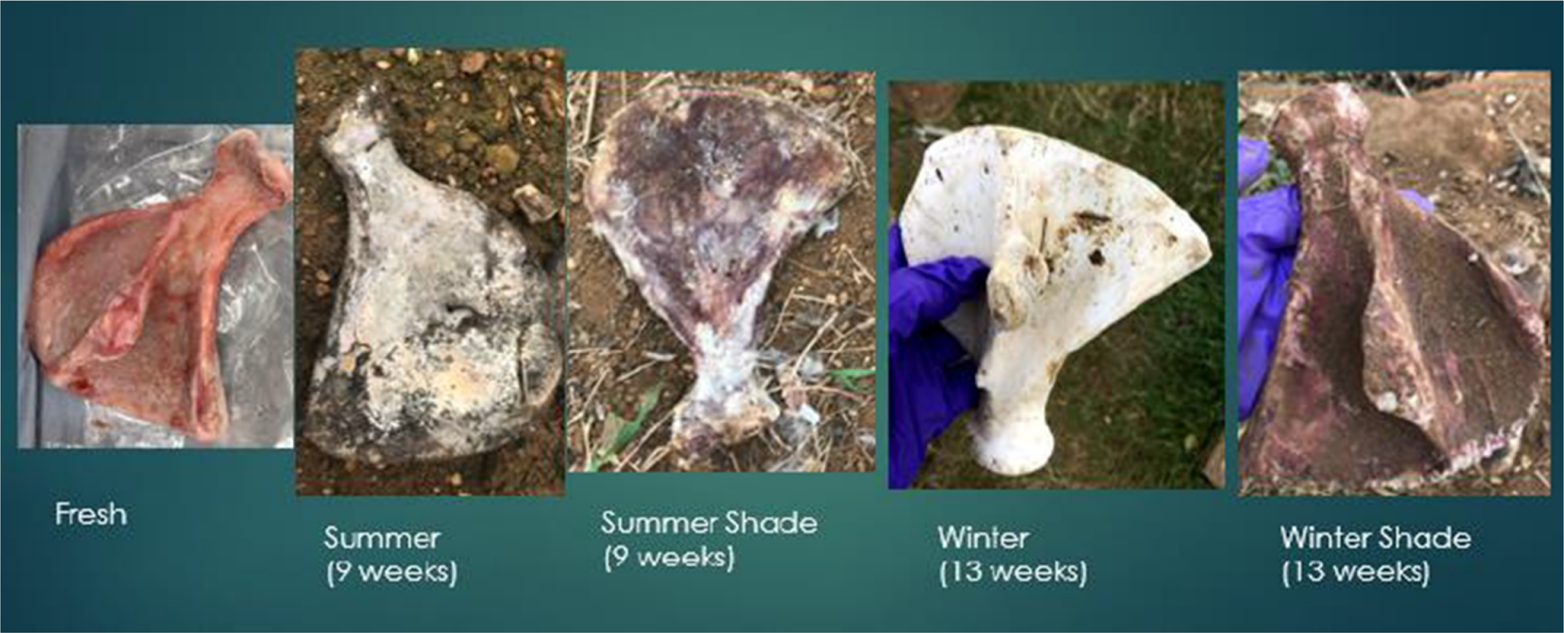
Colour changes to scapulae at different observations

**Fig. 3:**
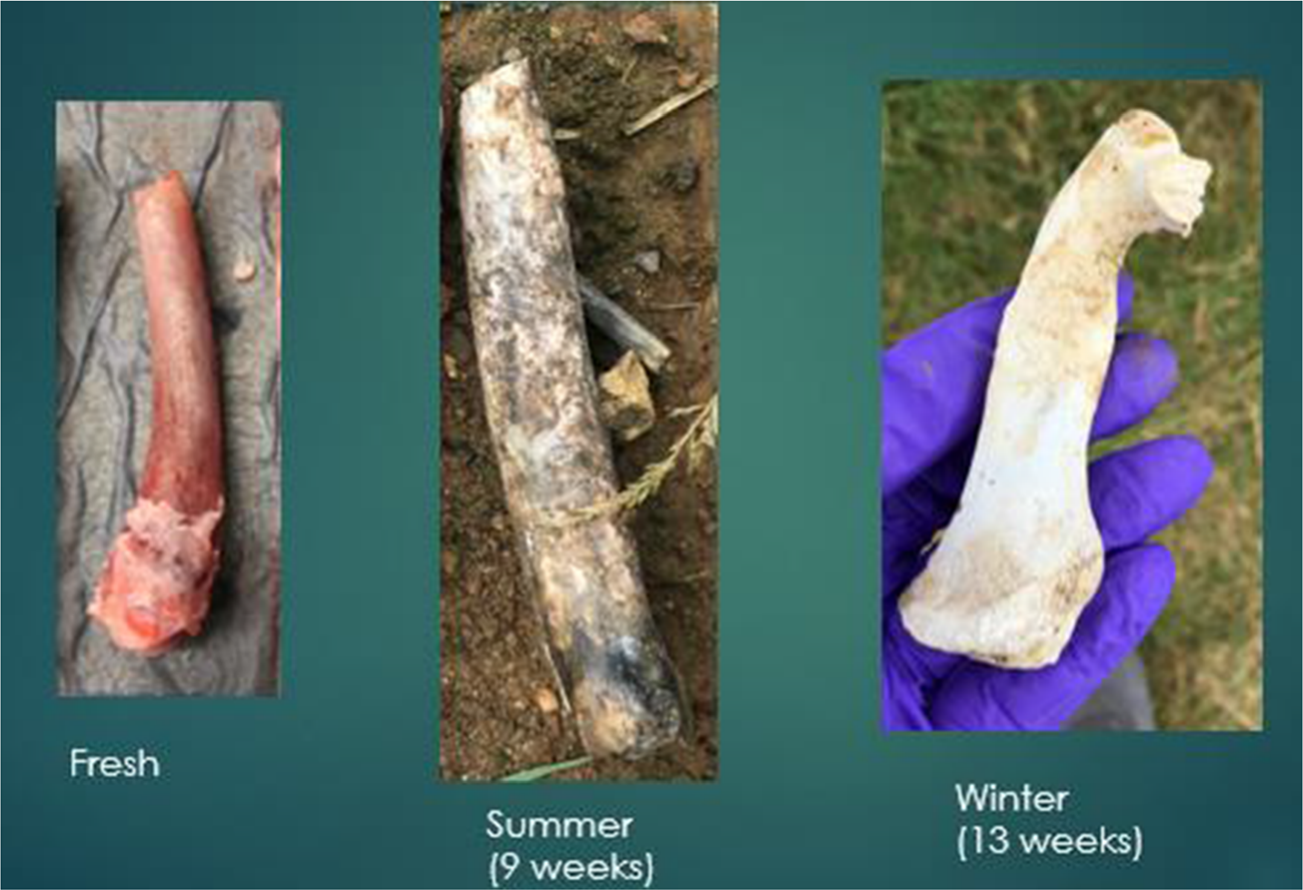
Rib colour changes at different observations

Bleaching was noticed in both summer and winter seasons on all three types of bone. The shaded samples did not bleach for either seasons (Figs. 1, 2, and 3).

**Fig. 4:**
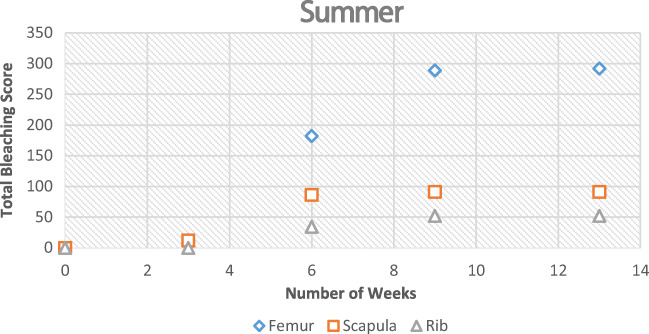
Observational bleaching scores for each bone type during the summer

Bleaching had started to occur at the 6-week interval when placed on an open field during the summer season. The section of bone with the highest scoring points was the anterior mid-shaft segment of the femur, the anterior side of the neck of the scapula, and the distal and proximal ends of the rib samples. At the 9-week interval, all samples were completely bleached for all three bone types. During the summer, the scores for the femur were the highest and had bleached the quickest (Fig. 4).

**Fig. 5:**
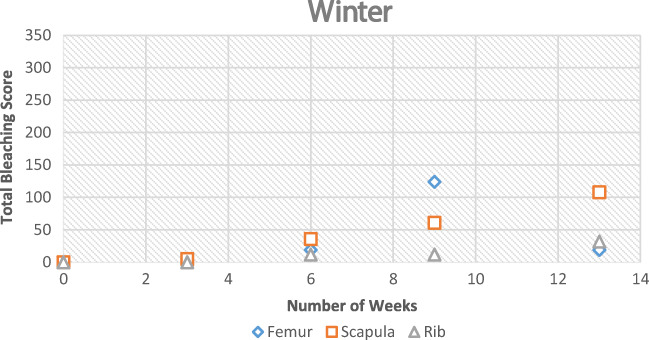
Observational bleaching scores for each bone type during the season of winter

**Fig. 6:**
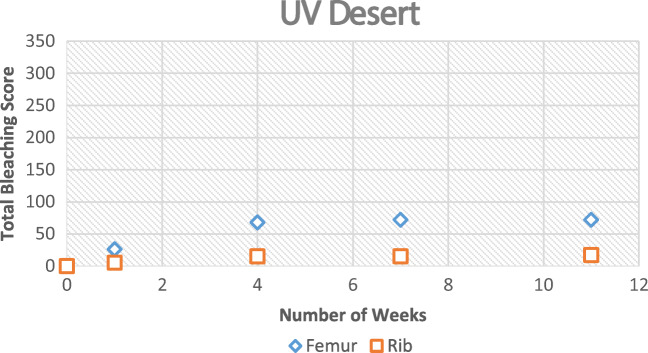
Observational bleaching scores for each bone type during the UV desert experiment

During winter, the process was very similar; however, it took longer to reach each stage (Figs. 5 and 6). The bleaching of the bone had started to begin at the 9-week interval. It was also noted that this started on the anterior mid-shaft section of the femur, the anterior area of the neck of the scapula, and the distal and proximal end of each rib. However, for the femur and ribs (except one sample), the colouring was of the natural cream bone colour whereas the scapula and one rib had fully whitened at week 13 (Table 1). The scapula samples appeared to bleach during the winter which was unexpected and appeared whiter than the other two types. The observational desert samples started to whiten after week 1 at the anterior mid-shaft section of the femur and the distal and proximal ends of the ribs; however, this did not progress after this initial week.

**Table 1:** Observational results for the time of bleaching for each bone type. First signs indicates that bleaching had started, and more than one area was affected. Unsuccessful refers to bone which had not bleached by the end of the season or by the 8th week in the desert environment experiment

Observational results		Femur	Scapula	Rib	Shaded
Summer	First signs	6 weeks	6 weeks	6 weeks	9 weeks
	Advanced bleaching	9 weeks	9 weeks	9 weeks	Unsuccessful
Winter	First signs	9 weeks	9 weeks	9 weeks	Unsuccessful
	Advanced bleaching	Unsuccessful	13 weeks	Unsuccessful	Unsuccessful
Desert	First signs	1 week	N/A	1 week	Unsuccessful
	Advanced bleaching	Unsuccessful	N/A	Unsuccessful	Unsuccessful

The UV levels were significantly reduced during the winter when compared with the summer. The winter season also experienced a significant amount of rain and snow which contributed to humidity levels. A percentage was calculated to show the prevalence of bleached bones during both seasons (Tables 2 and 3).

**Table 2:** The percentage of samples that had bleached by the end of the seasons

	Number of observational samples placed		Bleached		Bleached (%)	
	Summer	Winter	Summer	Winter	Summer	Winter
Femur	6	6	6	0	100	0
Scapula	5	6	4	3	80	50
Rib	5	6	3	1	60	17

**Table 3:** Average UV and ADD during both winter and summer seasons

	UV	ADD (°C)
Summer	5.11	16.54
Winter	1.00	4.87

The shaded environment of the open-field samples as well as the samples placed in a dark cupboard showed that no bleaching or whitening of the bone occurred in these experimental settings. The femur had started to whiten along the anterior and lateral mid-shaft area and the anterior side of the neck on the scapula but this was very minimal. The shaded and dark cupboard samples became dark red in colour during the first week and did not change colour thereafter. However, they did dry out and reduced in mass. All rib samples that were placed in the shade were scavenged.

There was a significant reduction in the weight of the samples at the end of the experiment. This also appeared to be more prominent in the winter months than the summer (Table 4).

**Table 4:** Mass reduction percentage figure for each experiment type where observations are the full-sized bones and fragmented samples

Sample type		Mass reduction (%)
Summer	Observational	27
	Analysis	24
Desert	Observational	48
	Analysis	18
Winter	Observational	52
	Analysis	48

Spectrophotometry was recorded at each interval for all the fragmented samples. As can be seen in Table 5 for the summer months, the L value which provides quantification for ‘lightness’ deceased, suggesting the bone was getting darker in colouration. The A value increased, suggesting a darker red pigmentation. The B value increased, showing a yellow pigmentation. The values for the winter samples were also in keeping with the observational findings: the L value increased showing a ‘lightening’ of colouration, the A value reduced showing a decrease in redness, and the B value increased showing an increase in yellow colouration. The desert sampled followed the same trend as the winter months with an increase in lightening, decrease in redness, and decrease in yellowing.

**Table 5:** Spectrophotometry results before and after sun exposure in each experiment type

Spectrophotometry	Before	After
Summer		
L	72.38 ± 1.6	66.25 ± 0.5
A	0.10 ± 0.2	1.31 ± 0.1
B	10.12 ± 1.2	13.80 ± 0.8
Desert		
L	59.83 ± 0.9	71.06 ± 0.7
A	7.53 ± 0.2	3.15 ± 0.7
B	21.20 ± 0.3	16.78 ± 0.7
Winter		
L	62.09 ± 0.5	64.49 ± 0.3
A	9.66 ± 0.4	2.73 ± 0.4
B	10.61 ± 0.4	19.31 ± 0.4

(L = lightness 0–100, 0 is black and 100 is white; A = red/green value, + 80 red and − 80 green; B = blue/yellow value, + 80 yellow and − 80 blue)

### Physicochemical properties

When analyzing the FTIR spectra, the crystallinity index had increased slightly after each experiment, especially in the summer samples. The mineral to organic ratio was calculated and showed a slight increase in both summer and winter but reduced for the UV desert experiment. The relative amount of carbonate was calculated, and this remained the same in the summer and desert experiment whilst it was reduced in the winter samples (Table 6).

**Table 6:** FTIR results for each experiment type. The control sample having no exposure, summer and winter having 12 weeks of exposure, and the desert samples having 8 weeks of exposure

FTIR	Crystallinity index	*P* value	Mineral to organic ratio	*P* value	Carbonate to phosphate	*P* value
Control	2.46 ± 0.008	-	2.35 ± 0.04	-	0.04 ± 0.0004	-
Summer	2.53 ± 0.004	0.15	2.76 ± 0.05	0.73	0.04 ± 0.0009	0.65
Desert	2.48 ± 0.002	0.00	2.34 ± 0.3	0.28	0.04 ± 0.0008	0.05
Winter	2.47 ± 0.005	0.76	2.79 ± 0.0003	0.00	0.03 ± 0.00009	0.53

A paired *t* test was performed using SPSS version 20 to explore whether these changes were statistically significant when analytical samples were compared with controlled samples. The CI and carbonate to phosphate were significantly greater for the desert samples, whilst the mineral to organic ratio was greater for the winter samples when compared with the control samples.

The Pearson correlation coefficient was calculated using the bleaching scores (observational sample data) against both UV and ADD data that was collected (Table 7). This showed a positive correlation. During the winter season, the UV levels appeared to be increasing with bleaching which correlated well with the scapula and rib samples. Towards the end of the summer experiment, the UV and ADD started to decrease in preparation for autumn. However, the results still showed a positive correlation, albeit small.

**Table 7:** Pearson’s correlation coefficient of observational bleaching scores against both UV and accumulated degree days for each experiment type. These values were calculated using SPSS 20

Pearson’s correlation (*r*)	Summer			Winter		
	Femur	Rib	Scapula	Femur	Rib	Scapula
UV	0.57	0.55	0.48	0.08	0.92	0.88
ADD	0.45	0.44	0.55	0.13	0.26	0.27

## Discussion

### Observation analysis

It was clear that bone mass reduced the longer the bone was exposed to UV which is commonly associated to the loss of water. This appeared to be more apparent when the bone was placed in a shaded area, as the bone would have been protected from rainfall. The bones that were placed in shade never bleached and remained a dark red/brown colour. This confirmed that without direct UV exposure, bones do not lighten in colour, similarly to buried bones. The bones that were sun-exposed during the summer started bleaching around the shaft of the bone initially by the 6th week and fully bleached by the 9th week. This was the case for all three bone types but whitening was more prominent in the femur and scapula than in the rib. Examining the UV and ADD data, there is a progression of bleaching the longer it is in an open area.

The percentage of bone that bleached in the summer was higher in the femur than any other bone type (Table 2). In winter, the percentage of bones that bleached were significantly lower than the percentage bleached in summer. This is consistent with a reduced UV exposure. However, this may be confounded by the level of scavenging that occurred to the femur samples during winter. In winter, the remaining three types of bone did start to bleach at 9 weeks. When examining the UV data for this period of time, it was much different than the summer records, and therefore, the process was much slower. Bleaching was different between the seasons. The bleaching that occurred in the summer was rapid and at surface level; whereas in winter, the bleaching depth appeared to be greater. The samples that were placed in the shaded area were not affected by bleaching during both seasons as expected.

A pattern was observed in the development of bone bleaching. The bleaching of the femora started at the anterior mid-shaft section and then progressed to the lateral and medial shaft, finishing at the epiphysis. On the scapula, bleaching started at the anterior neck and then to the supraspinatus fossa and infraspinatus fossa, before progressing onto the scapular spine and the remainder areas. The rib samples bleached first at the proximal and distal ends after which bleaching progressed to the mid-shaft area.

The desert experiment did not result in an amount of bleaching that was initially expected. The analysis samples did fully bleach whilst the observational samples did not fully bleach. The samples had only started to whiten at the external mid-shaft area and the ends of the ribs. This had the same effect as those samples that were placed in the dark cupboard. There was only a slight difference between the UV desert samples and the dark cupboard samples which was that the mid-shaft area was less bleached on those in the dark cupboard. It could be that the UV lighting equipment was not adequate enough to penetrate the bone material to bleach the samples in all areas as the bleaching progression came to a halt after the first week. The bone mass continued to reduce. The controlled desert simulation with no external weathering factors showed no increase in bleaching after the second interval of this experiment and this could have been because of low-cost lighting. The UV lighting was not as effective after the 3 weeks. If the UV lighting was up to standard consistently, the results may have been different. The samples placed in the dark cupboard that replicated shade had limitations of non-movement. In an actual desert setting if bones were left in the shade, they would still have the heat element involved from UV rays. As they were placed in a dark, cool place, it would have been expected that no bleaching would occur because there were no conditions for this to happen.

When analyzing L*A*B colour space on the bone fragments at three different points, the areas that were most bleached showed a high L value but these higher values were masked when an average was calculated. This may be due to the areas of the bones being more bleached than others. These results did not provide a measured value for bleaching as the results did not marry up with the observational findings. What was noticed however, was a change in the colouring related to the A and B values. There was no consistent increase in the L value that would give concrete support to this interpretation.

There was a positive correlation between the weather data and the bleaching scores. However, this cannot be interpreted as the only cause for bone bleaching due to the various taphonomic processes that would have contributed to these results, such as the microenvironment and other weathering factors [19–23]. As we know from Pokines and Higgs [15], bleaching occurs in  marine environments, and therefore, rainfall may have been a significant contribution to this. In addition to this, the colour of bleached bone is subjective and scoring may differ according to the observer and so these results are based solely on the subjective observational bleaching scores that were created.

Although UV radiation has been proven to contribute to bone bleaching, the rate at which bleaching occurs depends entirely on the combination of all taphonomic factors involved and further research on sun bleaching within the UK would assist in confirming that. There are other weather environments such as snowfall which may give different results. This can halt the bleaching process by a short period of time and so when comparing with the recent study in Florida, the bleaching rate would differ significantly [15, 24].

What is interesting with this experiment is that two sets of samples were placed in two different areas, only metres apart and when comparing the shaded sample against the sun exposed samples, there are clear differences between the colouring of the bone. It is visibly apparent that bleaching has occurred, which was progressive and noticeable at each stage of the process. This pattern was consistent for both seasons, and the only difference between the two seasons was that in the winter, the process was slower than summer which is likely to be due to the decrease in UV radiation.

Scavenging was a significant problem during the winter season particularly with the femora samples. Gnawing indents were visible on all of the samples and it is likely to have had an impact on the surface bone bleaching. It is possible that the bleaching was occurring sooner than recorded but the evidence of this was being destroyed by animal activity. During February 2018, the UK had a significant amount of storms and so sample movement increased between intervals. This would have interrupted bleaching due to re-positioning of the samples. Precipitation was also significant during the winter season which increased the moisture of the soil and samples. This would naturally slow down the bleaching process as the surface of the bone was often damp.

### Chemical analysis

The FTIR showed that there was a statistical significance between the control and experiment samples. The mineral to organic ratio for the winter samples and the crystallinity index for the desert analysis samples were significantly greater than the controls (*p* < 0.05). There was an increase in the mineral to organic ratio for the winter samples; however, no change was observed in the crystallinity index. This suggests that there was a reduction in the organic content of the samples but no change to the mineral crystal chemistry [24]. These results suggest the collagen within the bone had started to break down due the weathering conditions.

For the desert samples, the crystallinity index was significantly greater than the controls. This suggests the hydroxyapatite mineral crystals are significantly larger for bleached desert samples. The results would be suggestive of chemical changes occurring when bone is undergoing sun bleaching. As this experiment did not involve other weathering factors, it could suggest the UV exposure as the direct cause for this chemical change. It did not occur in the control or shaded samples where there was no UV exposure.

The aim of this research was to provide an insight into bone bleaching within a UK environment during the summer and winter seasons. Using a 0–5 scoring system of bleaching, quantitative data was achieved to determine a minimum PMI. This study showed that bleaching initiated at 6 weeks within the summer and fully bleached at week 9. In contrast, in the winter months bleaching initiated at 9 weeks, and a small percentage of the scapulae and ribs fully bleached at 13 weeks. Therefore, it is evident that the bleaching rates differ significantly depending on the season. L*A*B scores confirmed that colouration changes occurred but failed to provide a supportive pattern of lightening. In addition to this, when bone is covered (shaded) no bleaching occurred. This work is of significant importance within forensic anthropology and is the first experimental study published to consider bone bleaching within the UK.
